# High-quality physiology of *Alcanivorax borkumensis* SK2 producing glycolipids enables efficient stirred-tank bioreactor cultivation

**DOI:** 10.3389/fbioe.2023.1325019

**Published:** 2023-11-23

**Authors:** Tobias Karmainski, Marie R. E. Dielentheis-Frenken, Marie K. Lipa, An N. T. Phan, Lars M. Blank, Till Tiso

**Affiliations:** iAMB—Institute of Applied Microbiology, ABBt—Aachen Biology and Biotechnology, RWTH Aachen University, Aachen, Germany

**Keywords:** hydrocarbonoclastic bacteria, glycolipid, biosurfactant, acetate, hydrocarbons, alkanes, membrane aeration, bioremediation

## Abstract

Glycine-glucolipid, a glycolipid, is natively synthesized by the marine bacterium *Alcanivorax borkumensis* SK2. *A. borkumensis* is a Gram-negative, non-motile, aerobic, halophilic, rod-shaped γ-proteobacterium, classified as an obligate hydrocarbonoclastic bacterium. Naturally, this bacterium exists in low cell numbers in unpolluted marine environments, but during oil spills, the cell number significantly increases and can account for up to 90% of the microbial community responsible for oil degradation. This growth surge is attributed to two remarkable abilities: hydrocarbon degradation and membrane-associated biosurfactant production. This study aimed to characterize and enhance the growth and biosurfactant production of *A. borkumensis*, which initially exhibited poor growth in the previously published ONR7a, a defined salt medium. Various online analytic tools for monitoring growth were employed to optimize the published medium, leading to improved growth rates and elongated growth on pyruvate as a carbon source. The modified medium was supplemented with different carbon sources to stimulate glycine-glucolipid production. Pyruvate, acetate, and various hydrophobic carbon sources were utilized for glycolipid production. Growth was monitored *via* online determined oxygen transfer rate in shake flasks, while a recently published hyphenated HPLC-MS method was used for glycine-glucolipid analytics. To transfer into 3 L stirred-tank bioreactor, aerated batch fermentations were conducted using *n*-tetradecane and acetate as carbon sources. The challenge of foam formation was overcome using bubble-free membrane aeration with acetate as the carbon source. In conclusion, the growth kinetics of *A. borkumensis* and glycine-glucolipid production were significantly improved, while reaching product titers relevant for applications remains a challenge.

## 1 Introduction

Biosurfactants, secondary metabolites synthesized by diverse microorganisms, possess amphiphilic structures that decrease surface tension (Abdel-Mawgoud et al., 2010; Kubicki et al., 2019). These molecules exhibit various structural elements, including fatty acids, phospholipids, glycolipids, lipopeptides, lipoproteins, and polymeric biosurfactants (Desai and Banat, 1997). Their structural diversity contributes to various biological and physicochemical properties such as low critical micelle concentrations (CMC), strong surface tension reduction, metal ion chelation, bioactivity, and high tolerance to unfavorable pH values, temperatures, and ionic strengths (Santos et al., 2016; Schlebusch et al., 2023). In nature, biosurfactants play a crucial role in facilitating the utilization of hydrophobic substrates by microorganisms and can also contribute to the virulence of pathogens by facilitating cell lysis, as reported for *Pseudomonas aeruginosa* (Van Delden and Iglewski, 1998; Tripathi et al., 2018). Unlike synthetic surfactants, biosurfactants can be produced from renewable resources, exhibit lower toxicity, and are biodegradable (Poremba et al., 1991; Santos et al., 2016; Voulgaridou et al., 2021). So-called plant-derived or biobased synthetic surfactants are already available. They are produced from renewable carbon resources, mainly palm oil (Permadi et al., 2017). Palm trees are efficient and fast-growing plants, but their cultivation causes massive rainforest deforestation, negatively impacting biodiversity (Cazzolla Gatti and Velichevskaya, 2020). Furthermore, converting rainforests to palm tree plantations increases CO_2_ emissions (Cooper et al., 2020).

*Alcanivorax borkumensis* SK2 is an obligate hydrocarbonoclastic, Gram-negative, aerobic, halophilic, rod-shaped γ-proteobacterium known for its biosurfactant production. The complete sequencing of the *A. borkumensis* SK2 genome was published in 2006 (Schneiker et al., 2006), and subsequent genome sequencing of other *Alcanivorax* species followed (Lai and Shao, 2012a; Lai and Shao, 2012b; Lai et al., 2012; Fu et al., 2018; Sinha et al., 2021). The genome of *A. borkumensis* SK2 shows significant sequence similarities to *P. aeruginosa* and *Acinetobacter* sp. (Reva et al., 2008). The relatively small genome size of 3.12 Mbp indicates a highly specialized organism (Schneiker et al., 2006). *A. borkumensis* SK2 was initially isolated in 1992 from seawater near the island of Borkum (Passeri et al., 1992; Yakimov et al., 1998). It is naturally present in low abundance in unpolluted marine environments but substantially increases cell numbers following oil pollution. In those cases, it can constitute up to 90% of the microbial community involved in oil degradation (Golyshin et al., 2003; Harayama et al., 2004; Djahnit et al., 2019). As *A. borkumensis* is halophilic, many of its transport systems are sodium-dependent, enabling it to use the sodium gradient as an energy source for nutrition uptake, *e.g.*, sodium/alanine or sodium/sulfate symporters. The efflux of sodium ions, and thus the maintenance of the proton gradient across the membrane, is ensured by sodium/hydrogen exchangers, ATP-consuming sodium pumps, and by the exit of sodium *via* parts of the respiratory chain (Schneiker et al., 2006). An optimal NaCl concentration of 3%–10% is necessary for growth, and magnesium ions are needed to prevent cell lysis (Yakimov et al., 1998). This bacterium is known for two prominent characteristics: the ability to degrade aliphatic and branched hydrocarbons and the production of a glycolipid (Passeri et al., 1992; Abraham et al., 1998; Yakimov et al., 1998). Hydrophobic substrates like hydrocarbons hold the drawback of reduced bioavailability in aqueous environments due to low solubility (Eastcott et al., 1988). A common microbial strategy to overcome this barrier is the production of biosurfactants (Ron and Rosenberg, 2001).

Three main steps are involved in the degradation of alkanes in *A. borkumensis*. Firstly, the alkane is taken up and undergoes terminal oxidation, forming an alcohol (Rojo, 2009). This oxidation process can be facilitated by either AlkB1 monooxygenase or a second monooxygenase called AlkB2 (Sabirova et al., 2006b; Schneiker et al., 2006). Additionally, cytochrome P450(a), P450(b), and P450(c) can be present, which form an oxygenase system. The genes encoding these cytochromes are upregulated in the presence of isoprenoids and may participate in the oxidation of specific alkanes (Schneiker et al., 2006). Another monooxygenase known as AlmA potentially plays a role in the oxidation of *n*-tetradecane and pristane (Gregson et al., 2019). In the second step, the alcohol is further oxidized to a fatty acid *via* an aldehyde, possibly mediated by the alcohol dehydrogenase AlkJ and the aldehyde dehydrogenase AlkH (Schneiker et al., 2006; Gregson et al., 2019). Finally, the fatty acid is activated with CoA through the action of AlkK, an acyl-CoA synthetase. This enzymatic step makes the fatty acid accessible for ß-oxidation (Schneiker et al., 2006; Rojo, 2009). While *A. borkumensis* exhibits a remarkable hydrocarbon substrate range (C_5_-C_32_), its hydrophilic substrate range is limited. This limitation arises from the absence of genes encoding key enzymes, such as glucokinase, involved in glycolysis and the pentose phosphate pathway, as well as genes encoding proteins responsible for sugar transport (Schneiker et al., 2006). Consequently, *A. borkumensis* cannot utilize sugars as a carbon source (Yakimov et al., 1998).

**FIGURE 1:**
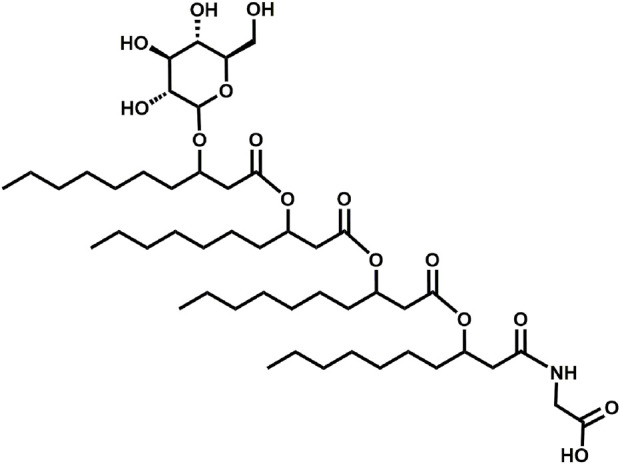
Basic structure of glycine-glucolipid of *A. borkumensis* SK2. The glycolipid consists of four 3-hydroxy fatty acids linked by ester bonds, glycosidically linked to the C_1_ atom of glucose, and linked to glycine *via* an amide linkage at the terminal fatty acid.

The glycine-glucolipid of *A. borkumensis* SK2 is a glycolipid and consists of four 3-hydroxy-fatty acids, with different chain lengths (C_6_-C_12_), linked to each other by ester bonds and glycosidcally linked to the C_1_-atom of glucose. The amino acid glycine is attached to the glycolipid at the terminal 3-hydroxy-fatty acid via an amide linkage (Figure 1) (Abraham et al., 1998; Cui et al., 2022; Lipphardt et al., 2023). Previous studies by Yakimov *et al.* (1998) suggested the presence of both a glycine-free extracellular glucolipid and a membrane-associated form of the glycine-glucolipid in *A. borkumensis* SK2. However, recent research has refuted the existence of the extracellular glycine-free form and revealed that the predominant form is biomass-associated without a glycine-free form being detected (Cui et al., 2022; Lipphardt et al., 2023). The glycine-glucolipid attached to the cell surface enhances cell surface hydrophobicity, leading to increased adherence of the bacterium to oil/water interfaces and improved bioavailability of hydrophobic carbon sources (Naether et al., 2013; Abbasi et al., 2018; Godfrin et al., 2018). This strategy of increasing cell surface hydrophobicity is also observed in other microorganisms, such as *Candida tropicalis*, *Acinetobacter calcoaceticus*, and *Rhodococcus erythropolis* (Kaeppeli and Fiechter, 1976; Rosenberg et al., 1980; de Carvalho et al., 2009). The complete biosynthesis pathway of the glycine-glucolipid in *A. borkumensis* SK2 has not been elucidated. However, the ABO_1783 and ABO_2215 genes have been identified as potential contributors to glycolipid production (Schneiker et al., 2006). These genes encode glycosyltransferases with similarities to rhamnosyltransferase B (RhlB) from *P. aeruginosa*, which is responsible for attaching the first sugar unit in rhamnolipid synthesis (Ochsner et al., 1995).

In microbial cultivation, the production of biosurfactants encounters specific challenges, one of which is the significant occurrence of foaming. Foaming in bioprocesses can adversely affect various aspects, including product quality, quantity, and productivity, and potentially lead to the loss of biocatalysts (Junker, 2007; Demling et al., 2020; Tiso et al., 2020). Furthermore, the accumulation of foam can pose risks such as obstructing sterile filters, which jeopardizes sterility and potentially causes reactor overpressure (Delvigne and Lecomte, 2010; Routledge, 2012; Anic et al., 2018). Various approaches can be employed to mitigate foam formation in fermentation processes, such as adding antifoaming agents or utilizing mechanical foam breakers. Alternative approaches to foam prevention include *in situ* liquid-liquid extraction (Demling et al., 2020; von Campenhausen et al., 2023), foam fractionation (Blesken et al., 2020; Koop et al., 2020; Oraby et al., 2023), defoamers as substrates (Sha et al., 2012; Bator et al., 2020), pressurized headspace aeration (Weiser et al., 2022), and bubble-free membrane aeration (Bongartz et al., 2021; Bongartz et al., 2023).

Despite *A. borkumensis* being primarily associated with bioremediation, limited efforts have been made toward the biotechnological production of its glycolipid. The initial characterization of this glycolipid dates back to 1992 when fermentation was performed as nitrogen-limited fed-batch cultivation in a 10 L stirred-tank bioreactor using 3% Mihagol-S (composed of C_14_ and C_15_ *n*-alkanes) as substrate. After 91 h, 40 g L^-1^ of *n*-alkanes were consumed, resulting in a titer of 1.7 g L^-1^ of glucolipid and a specific yield of 70 mg g_CDW_^-1^ (Passeri et al., 1992). Only the glucolipid consisting of four 3-hydroxy-fatty acids with different chain lengths and one glucose molecule was produced during this fermentation process (Passeri et al., 1992).

Here, we present growth and glycolipid production of *A. borkumensis* SK2. We used different online analytics for medium optimization. The focus was on macronutrients, such as phosphate and nitrogen, as well as on the optimal growth and production temperature and alternative hydrophilic or hydrophobic carbon sources. Furthermore, batch fermentation in a stirred-tank bioreactor for glycine-glucolipid production with *n*-tetradecane and acetate as the sole carbon sources were investigated. In addition, a membrane module was investigated for bubble-free aeration to prevent foam formation during fermentation.

## 2 Materials and methods

### 2.1 Bacterial strain and media

The bacterial strain *A. borkumensis* SK2 (DSM 11573) was used for all cultivation experiments (Yakimov et al., 1998). All buffer stock solutions were filter-sterilized (PES, Filtropur BT50, 0.22 µm, Sarstedt AG & Co. KG, Nümbrecht, Germany). All media were additionally supplemented with the respective carbon source (pyruvate, formate, acetate, propionate, lactate, citrate, succinate, ethylene glycol, terephthalic acid, glycerol, ethanol, methanol, *n*-dodecane, *n*-tetradecane, or *n*-hexadecane) in various concentrations (Table 1). All substrate stock solutions were filter-sterilized (organic acids: PES, Filtropur BT50, 0.22 μm, Sarstedt AG & Co. KG, Nümbrecht, Germany) (hydrocarbons: PTFE, CAMEO syringe filter, 0.22 μm, Carl Roth GmbH & Co KG, Karlsruhe, Germany).

**TABLE 1:** List of substrates used in this work.

Carbon source	Stock concentration [g L^-1^]	Working concentration [g L^-1^]
Pyruvate	80.0	4.92, 5, 10, 15, 20
Acetate	200	5, 10, 15, 20
Formate	200	5
Propionate	200	4.12, 5, 10, 15, 20
Lactate	200	5.03
Citrate	200	5.40
Succinate	200	4.96
Methanol	790	5
Ethanol	789	5
Glycerol	630	5
Ethylene glycol	62.0	5
Terephthalate	66.5	5
*n*-hexadecane	770	4.83
*n*-tetradecane	760	4.83
*n*-dodecane	750	4.84

Standard ONR7a medium (Dyksterhouse et al., 1995) based on the ionic composition of seawater was used in this work and contained (per L) 22.79 g NaCl, 11.18 g MgCl_2_ × 6 H_2_O, 3.98 g Na_2_SO_4_, 1.46 g CaCl_2_ × 2 H_2_O, 1.3 g TAPSO, 0.72 g KCl, 0.27 g NH_4_Cl, 89 mg Na_2_HPO_4_ × 7 H_2_O, 83 mg NaBr, 31 mg NaHCO_3_, 27 mg H_3_BO_3_, 24 mg SrCl_2_ × 6 H_2_O, 2.6 mg NaF, 2.0 mg FeCl_2_ × 4 H_2_O.

Modified ONR7a medium contained (per L) 22.79 g NaCl, 11.18 g MgCl_2_ × 6 H_2_O, 3.98 g NaSO_4_, 1.46 g CaCl_2_ × 2 H_2_O, 11.92 g HEPES, 0.72 g KCl, 2.0 g NH_4_Cl, 0.46 g NaH_2_PO_4_ × 2 H_2_O, 83 mg NaBr, 31 mg NaHCO_3_, 27 mg H_3_BO_3_, 24 mg SrCL_2_ × 6 H_2_O, 2.6 mg NaF, 2 mL trace elements (500x). Trace elements contained (per L) 5 g FeSO_4_ × 7 H_2_O, 2.5 g MnSO_4_ × H_2_O, 3.2 g ZnCl_2_, 0.2 g CoCl_2_ × 6 H_2_O, 0.36 g CuSO_4_ × 5 H_2_O, 0.1 g Na_2_MoO_4_ × 2 H_2_O, 6.37 g Na_2_EDTA × 2 H_2_O. For a medium containing organic acids as a carbon source, a 238.3 g L^-1^ HEPES stock solution with a pH of 7.0 was used, resulting in a start pH of 6.8, and for hydrocarbons as a carbon source, a 238.3 g L^-1^ HEPES stock solution with a pH of 7.8 was used, resulting in a start pH of 7.5.

### 2.2 Cultivation conditions

#### 2.2.1 Shake flask cultivation

For plate cultures, *A. borkumensis* SK2 was streaked from a cryoculture on a marine broth (MB) agar plate with 10 g L^-1^ pyruvate and incubated for 48–72 h at 30°C. For pre-cultures, the strain was first cultivated in 100 mL shake flasks in 10 mL modified ONR7a with 10 g L^-1^ pyruvate at 30°C and 200 rpm (shaking diameter: 50 mm) for 20–24 h, if not mentioned otherwise. The strain was cultivated in 500 mL shake flasks in 50 mL modified ONR7a with the respective carbon source for main cultures. The main culture was inoculated to a final optical density (OD_600_) of 0.1 or 0.2 and cultivated at 30°C at 300 rpm (shaking diameter: 50 mm) with a filling volume of 10% if not otherwise mentioned.

#### 2.2.2 BioLector cultivation

Online measurement of growth *via* scattered light was enabled by the BioLector microbioreactor (Beckmann and Coulter GmbH, formerly m2p-labs GmbH, Aachen, Germany). For this purpose, 48-well FlowerPlates (MTP-48-B) sealed with gas-permeable sealing foils (AeraSeal foils, Sigma Aldrich, St. Louis, Missouri, United States) were used with 1 mL culture volume. The temperature was set to values between 27°C–35°C at 1,000 rpm (shaking diameter: 3 mm).

#### 2.2.3 Growth Profiler cultivation

The Growth Profiler 960 enabled online growth measurement *via* a series of photos (green values) (EnzyScreen BV, Heemstede, Netherlands). To determine the correlation between OD_600_ and green value for *A. borkumensis* SK2, a calibration was carried out by measuring OD_600_ and green value for various dilutions of cells in 0.9% (w/v) NaCl in triplicates. For this purpose, a white 24-deep well plate with transparent bottom (SystemDuetz; Enzyscreen, B.V., Heemstede, Netherlands) was used with 1 or 1.5 mL culture volume. The temperature was set to 30°C at 225 rpm (shaking diameter: 50 mm).

#### 2.2.4 Transfer rate online measurement (TOM) cultivation

For the determination of the oxygen transfer rate (OTR) and carbon dioxide transfer rate (CTR), *A. borkumensis* cells were inoculated to an OD_600_ of 0.2 in 25 or 50 mL (5%–10% filling volume) of the modified ONR7a medium and 10 g L^-1^ pyruvate/acetate or 4.83 g L^-1^ *n*-alkane. Cultures were incubated in a TOM shaker (Kuhner, Birsfelden, Switzerland) with a 50 mm shaking diameter at 300 rpm and 30°C. The OTR and CTR were measured online in mmol L^-1^ h^-1^ in duplicates and averaged. The growth rates were determined from the exponential trend line at the steepest point of the OTR curve (log-lin plot).

#### 2.2.5 Stirred-tank bioreactor cultivation

All fermentations were performed in a 3.0 L glass stirred-tank bioreactor with the BioFlo120 fermentation system (Eppendorf, Hamburg, Germany). The DASware control software controlled the bioprocess (v 5.3.1.; Eppendorf, Hamburg, Germany). For online data acquisition, the bioreactor was equipped with a pH probe (EasyFerm Plus PHI K8 225, Hamilton, Bonaduz, Switzerland), dissolved oxygen (DO) probe (VisiFerm DO ECS 225, Hamilton, Bonaduz, Switzerland), and a Pt100 temperature sensor. Automatic foam control was implemented *via* a foam sensor and Antifoam 204 (Sigma Aldrich, St. Louis, Missouri, United States). The pH value of 7.3 was kept constant by automatically adding 4 M H_2_SO_4_/NaOH *via* peristaltic pumps. The DO was maintained above 30% by automatically increasing the stirring rate from 300 to 1,200 min^-1^. A constant gas flow of 24 or 32.4 L h^-1^ with sterile air was supplied *via* a supply air bottle with a ring sparger. Exhaust gas was dried with an exhaust gas condenser, and O_2_ concentration (X_O2_) and CO_2_ (X_CO2_) concentration were measured with BlueVary Sensors in combination with the BlueVis software (both BlueSens gas sensor GmbH, Herten, Germany). The agitation shaft was equipped with a six-blade Rushton turbine (∅ = 5.3 cm). A filling volume of 1.2 L was used. Modified ONR7a medium with acetate or *n*-tetradecane as carbon source was filled into the bioreactor and heated to 30°C before inoculation. The bioreactor was inoculated from a pre-culture to an OD_600_ of 0.2.

#### 2.2.6 Bubble-free fermentation with membrane aeration

The setup and parameters for temperature, pH, and inoculation for fermentation with membrane aeration are comparable to conventional fermentation described in section 2.2.5. Deviations are described here. A poly-4-methyl-1-pentene (PMP) hollow fiber membrane (Oxyplus, 3M, Neus, Germany) was used. The BT Membrane Module Static 2 L (BioThrust GmbH, Aachen, Germany) contained about 1,430 membrane fibers, with a total membrane fiber length of 163 m and 0.195 m^2^ membrane area. The basic structure of the module was made of polyamide (Bongartz et al., 2021). No foam sensor or antifoaming agent was used. The agitation shaft was equipped with two Rushton turbines and a pitched blade turbine. The filling volume was increased to 2 L. The membrane module was sterilized with 70% ethanol and installed in the autoclaved bioreactor under sterile conditions. A constant gas flow of 60 L h^-1^ and a constant agitation speed of 300 min^-1^ were applied. The DO setpoint of 30% was maintained by increasing the transmembrane pressure (TMP) from 0 to 0.3 bar and with a DO cascade from X_O2_ of 21%–100%.

### 2.3 Analytics

#### 2.3.1 Optical density measurement

Optical density was measured at 600 nm with a Ultrospec 10 cell density meter (Amersham Biosciences, Amersham, Great Britain), and ultrapure water was used as blank.

#### 2.3.2 Ammonium quantification

Filtered samples were diluted 1:20 with ultrapure water, and a dilution series from 400 mg L^-1^–6.3 mg L^-1^ with NH_4_Cl for calibration was prepared. 10 μL of the dilution series and the diluted samples were pipetted into a 96-well plate. Then 200 μL of a reagent solution consisting of 17.98 g L^-1^ with Na_3_PO_4_, 32 g L^-1^ sodium salicylate, and 0.5 g L^-1^ sodium nitroprusside were added to each well. Each well was mixed with 50 μL of 5% NaClO in the last step. The plate was incubated at room temperature for 10 min. The plate was shaken for 0.5 min and measured at 685 nm in a Synergy Mx monochromator-based multi-mode microplate reader and the Gen5 software (BioTek Instruments, Winooski, Vermont, United States).

#### 2.3.3 Cell dry weight determination

For the quantification of the cell dry weight (CDW), 1 mL of the fermentation broth was centrifuged at 4°C and 21,130 × *g* for 5 min. The supernatant was transferred to a new reaction tube for further analysis. The pellet was washed with 1 mL of ultrapure water and centrifuged under the same conditions. The supernatant was discarded. The pellet was resuspended with 1 mL ultrapure water and transferred into a glass vial, which was previously dried for 48 h and pre-weighed. The CDW was weighed after drying the sample at 65°C for 48 h.

#### 2.3.4 Extraction and purification of glycine-glucolipids and aglycones

For the glycolipid extraction, 800 µL of the culture broth were taken, and the pH value was adjusted to pH 3.0 with 1 M HCl. The samples were mixed with 800 µL ethyl acetate and shaken on a vortexer for 10 min. The samples were centrifuged in a Heraeus Pico 17 centrifuge (Thermo Scientific, United States) at 17,000 × *g* for 2 min. The upper phase was transferred into a 15 mL tube. The extraction was repeated two times. The organic phase was evaporated in a Scan Speed 40 speed vac (Scanspeed, Lynge, Denmark) at 800 min^-1^, 20°C, and 20 mbar for at least 3 h. 150 μL chloroform were added to the tube to purify the evaporated samples. A CHROMABOND SiOH silica gel column (200 mg/3 mL, 55 µm) (Macherey-Nagel GmbH & Co. KG, Düren, Germany) was conditioned with eight-column bed volumes of chloroform (2.4 mL). Then, the sample was transferred to the column and washed with 2.4 mL chloroform. The glycolipids were eluted into a new conical tube with 13.3 column volumes (4 mL) of acetone/isopropanol (9 + 1, v/v). The eluate consisting of glycolipids and acetone/isopropanol was evaporated under the same conditions as during the extraction. The evaporated samples were vortexed with 100 µL acetone/isopropanol (9 + 1, v/v), and filtered with a 0.22 μm regenerated cellulose membrane syringe filter (Phenomenex, Torrance, United States).

#### 2.3.5 Quantification of glycine-glucolipids and aglycones

Glycine-glucolipid concentration was measured using an Ultimate 3000 high-pressure liquid chromatography (HPLC) system with a Corona Veo charged aerosol detector (CAD) (Thermo Scientific, Waltham, Massachusetts, United States). The Nucleodur C18 Gravity column (150 × 3 mm, 3 µm particle size; Macherey-Nagel GmbH & Co. KG, Düren, Germany) was used. The column oven was heated to 60°C. The injection volume was 5 µL. A solution of 0.2% formic acid (A) and acetonitrile plus 0.2% formic acid (B) was used as the mobile phase at a flow rate of 0.633 mL min^-1^. The method lasted 46 min and included an analytical and an inverse gradient. The analytical gradient started with 24% A and 76% B for 0.5 min. Then the ratio of B was increased to 100% within 36 min. This ratio remained constant for 5 min. Then the ratio was changed to 24% A and 76% B within 0.5 min and was kept constant until the end of the method. The software Chromeleon (Version 7.2.10, Thermo Scientific, Waltham, Massachusetts, United States) calculated the inverse gradient in the mode ‘keep solvent composition’, resulting in a flow rate of 0.633 mL min^-1^. The inverse gradient (offset volume 778 µL) started with 100% B for 1.7 min. In the next step, the proportion of B was decreased to 76%, and A was increased to 24% within 36 min. The ratio of 24% A and 76% B was kept constant until 37.7 min, after which the ratio was changed to 100% B within 0.5 min and kept constant until the end of the measurement (Lipphardt et al., 2023). The term “glycolipids” hereafter relates to the resulting natural mixture containing glycine-glucolipids and a small fraction of the aglycones.

#### 2.3.6 Hydrophilic carbon source quantification

Acetate and pyruvate concentration were measured in an Ultimate 3000 HPLC system (Thermo Scientific, Waltham, Massachusetts, United States) using an isocratic method with 5 mM H_2_SO_4_ as a mobile phase and a flow rate of 0.5 mL min^-1^. To prepare samples for HPLC analysis of pyruvate and acetate, the supernatant (after centrifugation, 2 min, 17,000 × *g*) was filtered through a syringe filter (CA, Rotilabo syringe filter, 0.2 μm, Carl Roth GmbH & Co. KG, Karlsruhe, Germany). An injection volume of 5 µL was used. A Metab-AAC column (Ion exchange, 300 × 7.8 mm, 10 µm particle size; ISERA GmbH, Düren, Germany) was used for the separation. The column oven was heated to 40°C, and detection was performed with a UV detector at 210 nm. The method lasted 22 min.

#### 2.3.7 HPLC-UV/RI-MS^2^ method for parapyruvate identification

Other extracellular metabolites were identified on a Nexera UHPLC system (Shimadzu Corporation, Kyōto, Japan) with 0.2% formic acid as eluent, and the flow rate was 0.4 mL min^-1^. 5 μL of the sample were injected onto an Isera Metab-AAC 300 × 7.8 mm column (ISERA, Düren, Germany). The column oven was heated to 40°C. Afterward, the flow was divided into two directions with a split ratio of 1:10. The major part of the samples was measured with a RID-20A refractive index detector and an SPD-40 UV detector at 210 nm (Shimadzu Corporation, Kyōto, Japan). The rest was analyzed with a triple quadrupole mass spectrometry (MS) 8060 (Shimadzu Corporation, Kyōto, Japan). The mass spectrometric parameters were: electrospray ionization (ESI) negative mode, desolvation line temperature: 250°C; nebulizer gas flow: 3 L min^-1^; heat block temperature: 400°C; other parameters were optimized automatically by auto-tuning (Phan et al., 2023). Product ion scan mode (*m/z* 30–300) was applied for precursor ions *m/z* 87 and *m/z* 175.2 with a collision energy of 10 V.

## 3 Results

High-quality physiology experiments of *A. borkumensis* SK2 are challenging as often poor growth is observed, biomass determination in cultivations with hydrophobic hydrocarbons is difficult, and the lack of appropriate analytics hampers determination of biosurfactant production. Here, we used an arsenal of techniques to provide insights into the physiology of *A. borkumensis* SK2.

### 3.1 Online growth monitoring shows nutrient limitations in ONR7a medium

**FIGURE 2:**
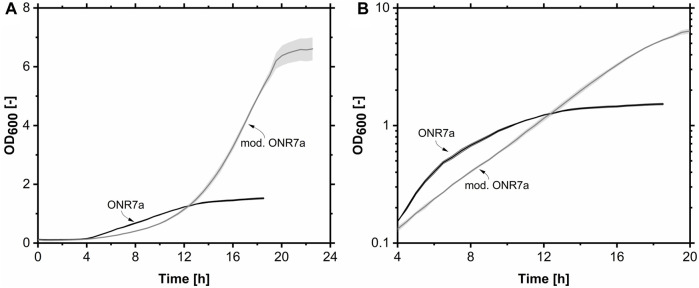
Growth Profiler cultivation of *A. borkumensis* SK2 on pyruvate in ONR7a and modified ONR7a media. **(A)** A lin-lin plot of the growth curves. **(B)** A log-lin plot of the growth curves between 4 and 20 h (same data). Error bands indicate deviation from the mean (n = 4). Cultivation conditions: ONR7a or modified ONR7a medium, 24-well white plate, N = 225 rpm, T = 30°C, OD_start_ = 0.1, V_L_ = 1.5 mL, 10 g L^-1^ pyruvate.

A defined growth medium is essential for secondary metabolite production like biosurfactants, as it allows fed-batch cultivation, where often second substrate limitations or specific carbon-to-nitrogen ratios are used (Invally and Ju, 2020; Merkel et al., 2022). In order to establish biosurfactant production, the growth behavior of *A. borkumensis* SK2 was investigated in the published ONR7a medium (Dyksterhouse et al., 1995) and the modified (mod.) ONR7a medium with 10 g L^-1^ pyruvate as carbon source in the Growth Profiler (Figure 2). To briefly describe the optimization, the buffer was exchanged from 5 mM TAPSO to 50 mM HEPES because the pH could not be maintained below 8.4 even at low cell densities. In addition, phosphate and nitrogen concentrations were increased 5.3- and 7.4-fold, respectively, as they were not sufficiently present in the original medium for extended exponential growth. Moreover, we added a trace element solution, although with only a slightly increased growth rate in the batch culture. We would argue that trace element limitation often occurs in fed-batch processes at higher cell densities. The individual optimization steps are shown in the Supplementary Material (Supplementary Figures S1, S2).

The growth on the two media was compared using pyruvate as the sole carbon and energy source (Figure 2A). On the ONR7a medium, growth ceased after 12 h. In comparison, the OD_600_ on the mod. ONR7a medium continued to increase after 12 h, reaching a maximum OD_600_ of 6.6, which was 4.4-fold higher, with a final pH of 8.3 after 20 h (Figure 2A). The literature medium supported a higher μ_max_ of 0.49 h^-1^ compared to the modified ONR7a medium of 0.26 h^-1^. However, only in a short period after that the growth rate reduced to 0.19 h^-1^. The increasing pH value may have an inhibitory effect on the cells. A constant μ_max_ of 0.26 h^-1^ was observed for the mod. ONR7a medium throughout the cultivation.

The optimal temperature for the process was investigated next to improve growth and glycolipid production further.

### 3.2 Thermal optimum for growth and glycolipid production

**FIGURE 3:**
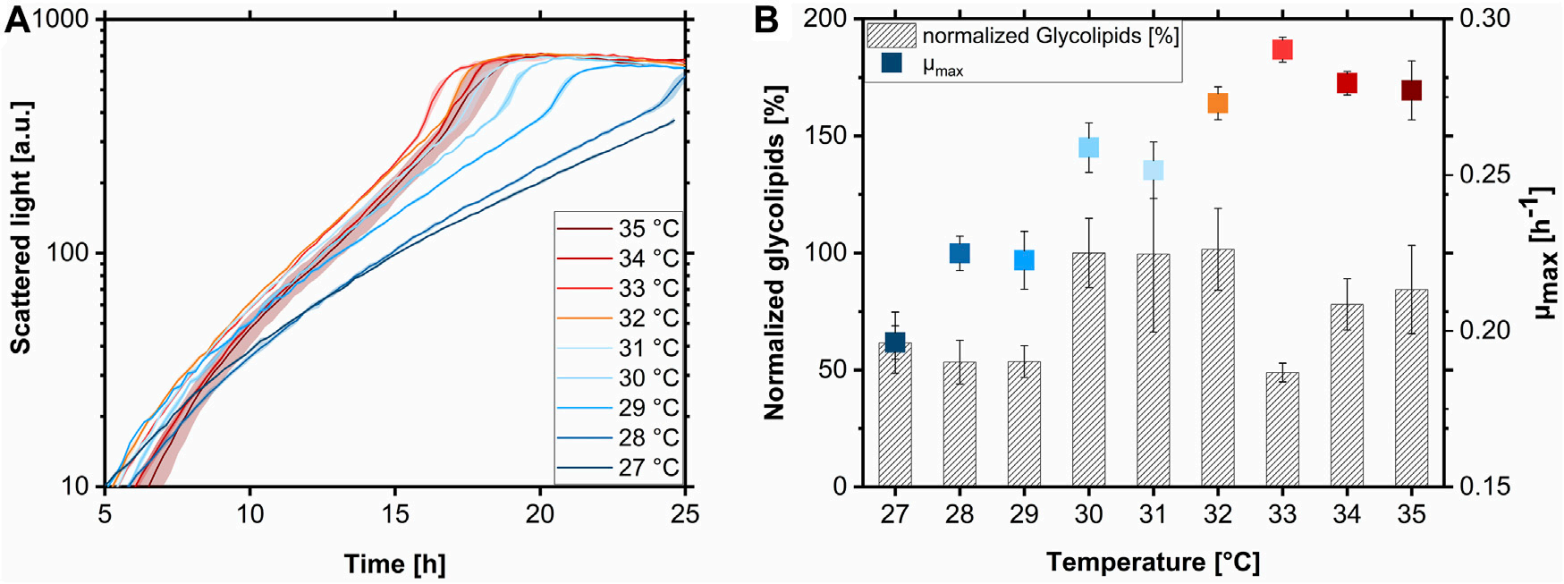
Growth behavior and glycolipid production of *A. borkumensis* SK2 at various temperatures with pyruvate. **(A)** BioLector backscatter growth curves at different temperatures, **(B)** growth rates (µ_max_), and normalized glycolipid production of *A. borkumensis* SK2 at temperatures between 27°C–35°C. Error bands/bars indicate deviation from the mean (n = 4 for 31°C–35°C and n = 7 for 27°C–30°C). Normalized glycolipids: the peak area of glycolipids produced per biomass at 30°C was set to 100%. All other amounts were set in relation to this value. Cultivation conditions: modified ONR7a medium, BioLector 48-well FlowerPlate (MTP-48-B), T = 27°C–35°C, N = 1,000 rpm, OD_start_ = 0.1, V_L_ = 1.0 mL, 10 g L^-1^ pyruvate.

Yakimov et al. (1998), the discoverers of the organism, reported the optimal growth temperature of *A. borkumensis* SK2 to be between 20°C and 30°C. However, no growth curves or growth rates are presented in the study. Growth and glycolipid production were investigated between 27°C and 35°C to fill these gaps using online analytics. The BioLector cultivation was carried out in mod. ONR7a medium containing 10 g L^-1^ pyruvate. During cultivation below 30°C, *A. borkumensis* SK2 showed decreased μ_max_ between 0.20 and 0.22 h^-1^. During growth at 30°C, the standard cultivation temperature, a μ_max_ of 0.26 h^-1^, was determined. When grown at higher temperatures, μ_max_ increased until it peaked at 33°C with a μ_max_ of 0.29 h^-1^. With further temperature increase, μ_max_ decreased slightly to 0.28 h^-1^ at 35°C (Figures 3A,B). The term “glycolipids” hereafter relates to the resulting natural mixture containing glycine-glucolipids and a small fraction of the aglycones.

Glycolipid titers were presented normalized here because, first, the cultures were harvested at different time points and different final biomass concentrations, and second, no calibration for the HPLC-CAD of glycolipids for absolute quantification was available at the time. Comparing normalized glycolipid production (the peak area of glycolipids produced per biomass at 30°C was set to 100%), there seemed to be a decreased production at temperatures below 30°C with values between 53%–61% (Figure 3B). Reduced biosurfactant productivity at lower temperatures was already observed with the Gram-negative bacterium *P. aeruginosa* PA01 (Müller et al., 2011). This reduced glycolipid production goes along with the low μ_max_ observed at these conditions. Between 30°C and 32°C, the glycolipid production was higher and reached a plateau. The lower value at 33°C was probably caused by a time delay in harvesting the cultures. The glycolipid remained in the biomass, being membrane-associated and not secreted into the supernatant (Abraham et al., 1998; Cui et al., 2022). Therefore, the lower glycolipid production at 33°C is not due to product degradation or uptake by *A. borkumensis* but because the cells stick to the MTP well wall and can no longer be rinsed off. With higher temperatures of 34°C and 35°C, slightly lower glycolipid production of 78%–84% was determined, along with the decrease of μ_max_ at temperatures above 33°C (Figure 3B).

The temperature spectrum for ideal growth and glycolipid production of *A. borkumensis* SK2 is proposed to be between 30°C and 33°C, with a presumable optimum at 33°C obtaining a μ_max_ of 0.29 h^-1^. Even though the glycolipid production at 33°C was not comparable due to experimental issues, the product formation is assumed to be at least as high as at temperatures between 30°C and 32°C. This high temperature optimum is surprising for a marine bacterium since the average temperature in the surface water in the North Sea is approximately 11°C. With an optimized medium and temperature established, growth kinetics on pyruvate in shake flasks were determined.

### 3.3 Pyruvate is not an ideal hydrophilic carbon source

**FIGURE 4:**
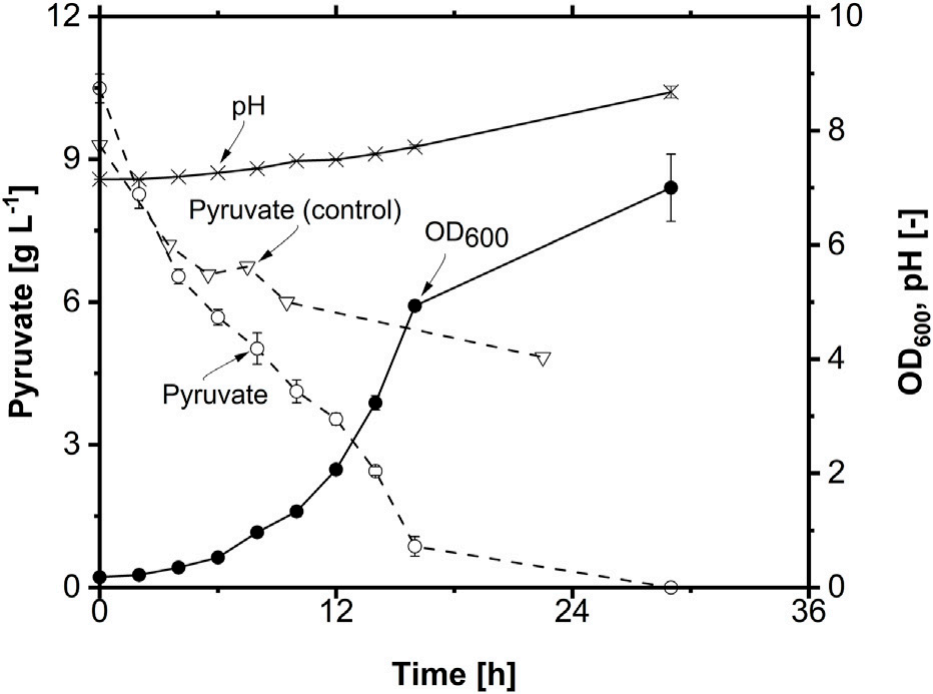
Physiology of *A. borkumensis* SK2 with pyruvate as the sole carbon source in shake flask. Pyruvate concentration in an abiotic control shake flask is also shown (Pyruvate (control) without inoculum) (n = 3). Cultivation conditions: modified ONR7a medium, 500 mL shake flask, T = 30°C, N = 300 rpm, OD_start_ = 0.1, V_L_ = 50 mL, 10 g L^-1^ pyruvate.

Subsequently, the MTP cultivation conditions were transferred to the shake flask scale for high-quality physiological analyses, including investigating growth behavior and substrate uptake. The shake flask cultivation was performed with mod. ONR7a medium containing 10 g L^-1^ pyruvate at 30°C and 300 rpm. A control shake flask, which was not inoculated, was also included. The growth curve progressed exponentially with a μ_max_ of 0.25 h^-1^, a final OD_600_ of 7.0, and a pH of 8.6 comparable to the MTP cultivations (Figure 4). The shape of the pyruvate concentration curve appears to be non-classical because the curve should be inversely proportional to the growth curve. When examining the pyruvate concentration in the control shake flask, it decreases, indicating that abiotic factors lead to pyruvate degradation. Using HPLC-MS for quantification of molecules in the medium (Supplementary Figure S3), a new peak was observed, the dimer of pyruvate, namely, parapyruvate. Analyzing the sample with MS in negative ion mode, the dominant ions were *m/z* 87 and *m/z* 175.2. A product ion scan was conducted for these two ions (Supplementary Figures S3A,B,C) for better identification. The results confirmed that *m/z* 87 and *m/z* 175.2 were the precursor ion [M-H]^-^ of pyruvate and parapyruvate, respectively. It was previously reported that pyruvate spontaneously dimerizes to parapyruvate under alkaline conditions (Chang et al., 2018).

Due to the instability of pyruvate in the cultivation medium, assessment of the carbon source concentration is impossible. Without quantification of the substrate, many process-characterizing parameters, like the substrate uptake rate or product-to-substrate yield, cannot be determined; hence, the cultivation cannot be adequately characterized, and the investigation of the impact of other parameters on the cultivation is impossible. Furthermore, pyruvate is also a costly carbon source. Thus, in order to select a suitable carbon source for high-quality physiology, other hydrophilic substrates were tested as the sole carbon source for the growth of *A. borkumensis* SK2.

### 3.4 Expansion of the hydrophilic substrate spectrum

**FIGURE 5:**
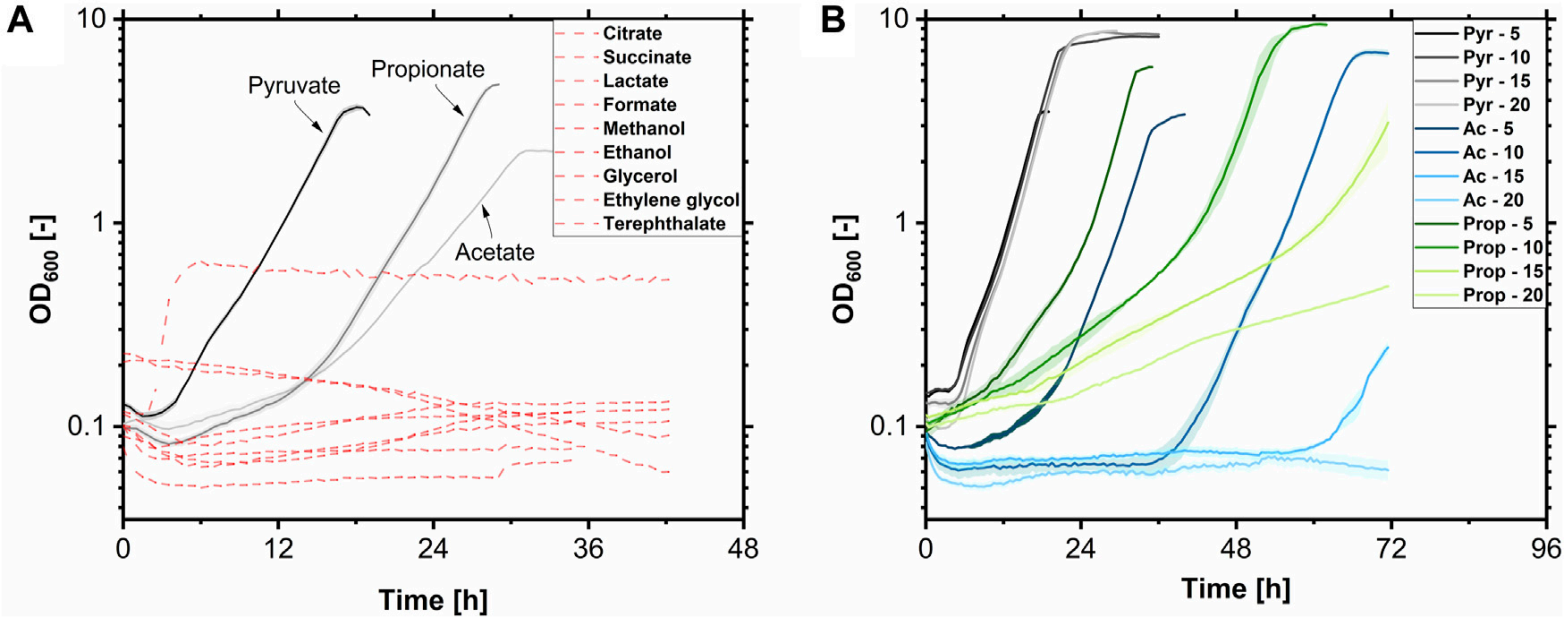
Growth of *A. borkumensis* SK2 on hydrophilic carbon sources. **(A)** Test for growth on 0.17 Cmol L^-1^ pyruvate, acetate, propionate, citrate, succinate, lactate, formate, methanol, ethanol, glycerol, ethylene glycol, and terephthalate. **(B)** Test for substrate inhibition at different pyruvate (Pyr), acetate (Ac), and propionate (Prop) concentrations (5–20 g L^-1^). Error bands indicate deviation from the mean (n = 4) **(A)** or range (n = 2) **(B)**. Cultivation conditions: modified ONR7a medium, Growth Profiler, white 24-well plate, N = 225 rpm, T = 30°C, OD_start_ = 0.1, V_L_ = 1.0 mL.

Different substrates were tested in the Growth Profiler in a 24-well MTP at 225 rpm and 30°C with a filling volume of 1.0 mL regarding growth to characterize the hydrophilic substrate spectrum of *A. borkumensis* SK2. Pyruvate was used as a reference. Substrates were used in equal Cmol concentrations of 0.17 Cmol L^-1^. Of the tested substrates (Figure 5A), only pyruvate, acetate, and propionate were suited as the sole carbon and energy source for *A. borkumensis*. While on terephthalate, a change in turbidity was monitored after 5 h. This did not signal growth but rather substrate precipitation. No growth was observed on citrate, succinate, lactate, formate, methanol, ethanol, glycerol, ethylene glycol, and terephthalate after 42 h. Yakimov *et al.* (1998) reported that *A. borkumensis* SK2 could utilize formate for growth; however, no growth curve was shown nor described which formate concentration was used. Formate is already toxic at low concentrations for many microorganisms. It cannot be used for growth by many microorganisms but only to detoxify by a formate dehydrogenase, thereby regenerating redox equivalents. Therefore, it can be used as a co-substrate (Zobel et al., 2017). The 0.17 Cmol L^-1^ formate might be a too high concentration, but lower concentrations make productive biotechnological processes cumbersome. When propionate or acetate were used as substrates, the lag phase was extended (15 h), indeed indicating weak acid sensitivity by *A. borkumensis*. The µ_max_ was 0.25 h^-1^ for pyruvate and propionate. The µ_max_ with acetate was 0.4-fold lower at 0.16 h^-1^. Subsequently, the influence of the substrate concentration of the suitable molecules on growth was tested (Figure 5B). Therefore, *A. borkumensis* SK2 was cultivated with 5, 10, 15, and 20 g L^-1^ of pyruvate, acetate, or propionate, respectively. While high pyruvate concentrations did not affect growth, lag phases increased with higher concentrations of acetate or propionate. Within 70 h, growth was still observed at concentrations of 15 g L^-1^ acetate and 20 g L^-1^ propionate, with a slight decrease in µ_max_.

Since acetate is a sustainable carbon source that can be produced from lignocellulose or syngas, acetate was selected for closer examination. Acetate is converted to acetyl-CoA *via* acetyl-CoA synthetase (AcsA) and is fed directly into central carbon metabolism *via* glyoxylate shunt (CCM). Acetyl-CoA is the direct precursor molecule to *de novo* fatty acid synthesis, which is required to produce the glycolipid. Growth and glycolipid production on acetate was compared to growth on hydrophobic carbon sources and pyruvate as controls.

### 3.5 In-depth characterization of glycolipid production shows superiority of hydrophobic substrates

*A. borkumensis* SK2 was cultured in the transfer rate online measurement (TOM) incubator to observe growth and glycolipid production. This device can monitor the oxygen transfer rate (OTR) in shake flasks and thus was essential for monitoring growth, as the emulsion formation on hydrophobic substrates makes turbidity measurements obsolete (Demling et al., 2020).

**FIGURE 6:**
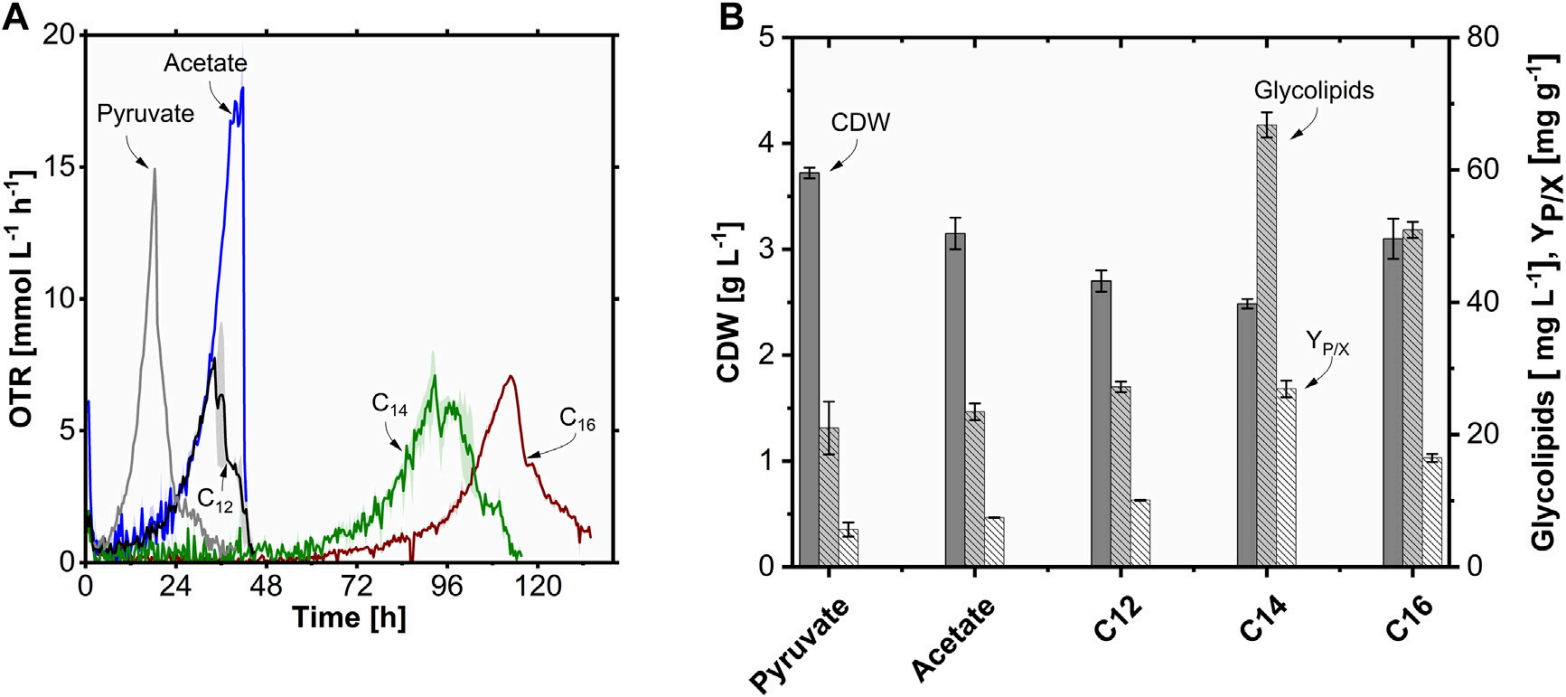
Comparison of growth and glycolipid production of *A. borkumensis* SK2 on five different carbon sources in shake flask cultivations. **(A)** Time course of oxygen transfer rate (OTR), and **(B)** cell dry weight (CDW), glycolipid concentration, and product-to-biomass yield (Y_P/X_) at the end of the cultivation. Error bands/bars indicate deviation from the mean (n = 2). Cultivation conditions: modified ONR7a medium, 500 mL TOM shake flask, T = 30°C, N = 300 rpm, OD_start_ = 0.2, V_L_ = 25–50 mL, 0.34 Cmol L^-1^ substrate.

In the TOM experiments (Figures 6A,B), growth and glycolipid production with an equimolar amount of carbon (0.34 Cmol L^-1^) of the substrates pyruvate, acetate, *n*-dodecane (C_12_), *n*-tetradecane (C_14_), and *n*-hexadecane (C_16_) were compared. The OTR of the pyruvate culture increased exponentially after a short lag phase with a µ_max_ of 0.25 h^-1^ to an OTR_max_ of 14.9 mmol L^-1^ h^-1^, which was reflected in a high CDW of 3.7 g L^-1^. For the acetate culture, the µ_max_ of 0.16 h^-1^ was 0.4-fold lower compared to pyruvate, which is consistent with the microtiter plate scale cultivation. However, the OTR_max_ of 18.0 mmol L^-1^ h^-1^ was 1.2-fold higher than with pyruvate. This higher OTR_max_ is probably due to the higher degree of reduction (DoR) of acetate compared to pyruvate (4.0 vs. 3.3). Pyruvate is more oxidized, and thus less oxygen per hour was required despite the higher µ_max_. The CDW on acetate was with 3.2 g L^-1^ slightly lower compared to cultivation with pyruvate. Interestingly, the lag phase increased while the µ_max_ decreased with increasing chain length of the hydrophobic substrates. While the µ_max_ and lag phase were invariant for C_12_ compared to acetate, the lag phase was significantly prolonged with C_14_ and C_16_ at 64 h and 82 h, respectively, and the µ_max_ with 0.09 h^-1^ for C_14_ and C_16_ by 0.4-fold decreased. The OTR_max_ were comparable among the hydrocarbons at about 7.0–7.8 mmol L^-1^ h^-1^. The CDW was also in a similar range for the *n*-alkanes with 2.7 g L^-1^ (C_12_) to 3.1 g L^-1^ (C_16_). *A. borkumensis* produced the highest glycolipid titer of 66 mg L^-1^ with C_14_ as a carbon source with a 3.2-fold increase compared to pyruvate with 21 mg L^-1^, which is also reflected in the highest product-to-biomass yield (Y_P/X_) of 27 mg g^-1^. In contrast to organic acids, *n*-alkanes (DoR >6) are highly reduced substrates. Furthermore, the medium-chain length fatty acid intermediates of the *n*-alkane catabolism (Rojo, 2009; Cui et al., 2022) could theoretically also be used as direct precursors for glycolipid production. Furthermore, the hydrophobic substrates might stimulate glycolipid production. This is followed by the glycolipid titers with C_16_ and C_12_ with 51 mg L^-1^ and 27 mg L^-1^, respectively. Cultivation on acetate was highly comparable to pyruvate with 23 mg L^-1^ glucolipid.

In summary, glycolipid production was highest with hydrophobic carbon sources (highest with C_14_). For closer examination, batch fermentations in stirred-tank bioreactors were carried out to maintain constant pH and to improve emulsion formation.

### 3.6 Cultivation in stirred-tank bioreactors increases the bioavailability of hydrophobic carbon sources

**FIGURE 7:**
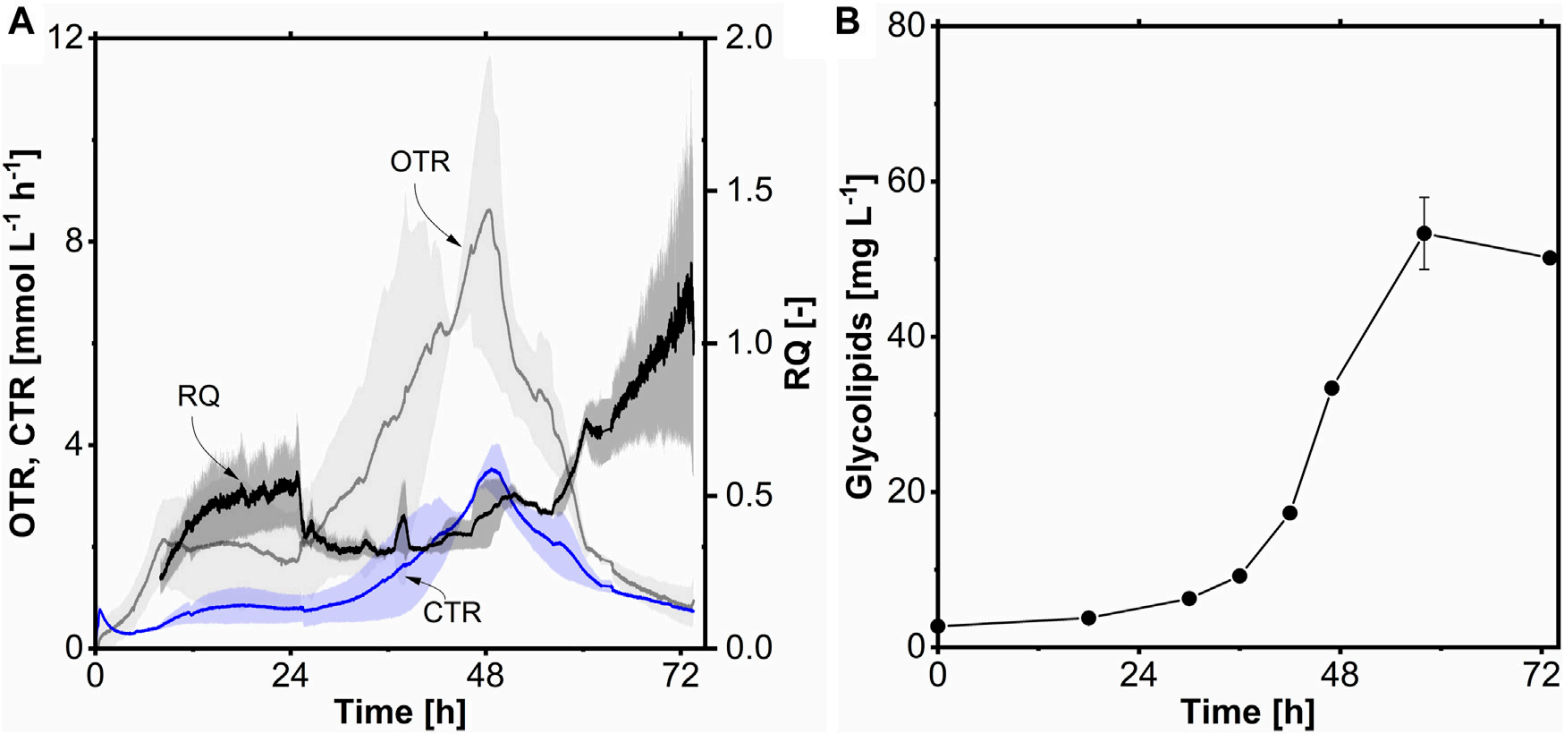
Batch fermentation of *A. borkumensis* SK2 with *n*-tetradecane as carbon source. **(A)** Time course of oxygen transfer rate (OTR), carbon dioxide transfer rate (CTR), and respiratory quotient (RQ); **(B)** glycolipid concentration course. Error bands/bars indicate deviation from the mean (n = 2). Cultivation conditions: modified ONR7a medium, 3 L stirred-tank bioreactor, T = 30°C, pH = 7.3, N = 300–1,200 min^-1^ (cascaded), DO = 30%, F_Air_ = 24 L h^-1^, OD_start_ = 0.2, V_L_ = 1.2 L, 4.83 g L^-1^ *n*-tetradecane.

Batch fermentation (Figure 7) was started with 4.83 g L^-1^ (0.34 Cmol L^-1^) *n*-tetradecane (C_14_), as C_14_ showed the highest glycolipid production in the shake flask experiment. A CDW time course could not be determined because the C_14_ interferes with the CDW determination as it attaches to the cells and thus distorts the weight. A maximum OTR of 8.6 mmol L^-1^ h^-1^ with a µ_max_ of 0.09 h^-1^ was reached, which is comparable to the shake flask experiment (Figure 7A). Interestingly, the lag phase was only 24 h long and 2.5-fold shorter than in the shake flask. This shorter lag phase is probably due to the mixing in the stirred tank reactor, which disperses the C_14_ more efficiently than in the shake flask, resulting in smaller oil droplets and higher surface area. The RQ is the ratio of CO_2_ produced (CTR) and O_2_ consumed (OTR). During growth on reduced substrates (DoR >4), the RQ is < 1, and when it grows on oxidized substrates (DoR <4), the RQ is > 1. The RQ was between 0.4 and 0.6 until the end of fermentation, indicating that *A. borkumensis* was growing on a highly reduced substrate, which is the case for C_14_ (DoR >6). Strong biofilm formation was observed on the vessel and internal installations during fermentation. Furthermore, flocculation of cells also occurred (Supplementary Figure S4). Therefore, the glycolipid titer of 53 mg L^-1^ was probably 0.2-fold lower than in the shake flask with C_14_ as the carbon source because of the underrepresentation of biomass (Figure 7B). Another reason for the lower glycolipid titer could be that sampling removed less or more oil phase from the reactor because the reactor is not ideally mixed. This can change the substrate concentration since not the same water and organic phase ratio is always taken from the reactor. With C_14_ as the sole carbon source, a product-to-substrate yield (Y_P/S_) of 155 mg Cmol^-1^ was obtained. Furthermore, a space-time yield of 1 mg L^-1^ h^-1^ was reached, which is 1.7-fold higher compared to shake flask cultivation.

In summary, stirred-tank bioreactor fermentation with a hydrophobic substrate showed improved growth and product formation kinetics compared to shake flask cultivations. Likely, the improved emulsification of the substrate leads to overcoming of mass transfer limitations between the hydrophobic and water phase and, finally, from the water phase to the cells. However, the fermentation showed that the process with oils is much more challenging to handle (sampling), and many process parameters, such as biomass, substrate concentration, or parameters based on optical measurements, such as photometric assays for macronutrients, are challenging to measure. Subsequently, the focus was placed on acetate as carbon source, as it is hydrophilic and performs better in terms of sustainability, as C_14_ is petroleum-based.

### 3.7 Hydrophilic substrates facilitate high-quality physiology in stirred-tank bioreactors

**FIGURE 8:**
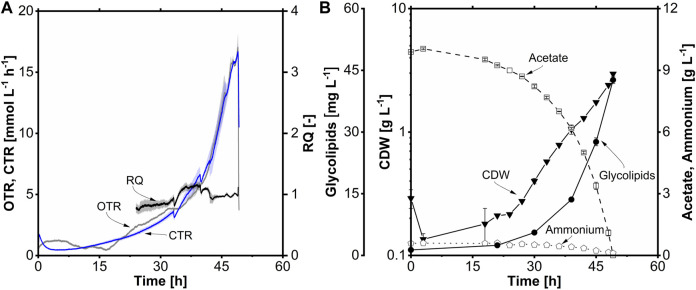
Βatch fermentation of *A. borkumensis* SK2 with acetate as carbon source. **(A)** Time course of oxygen transfer rate (OTR), carbon dioxide transfer rate (CTR), and respiratory quotient (RQ); **(B)** cell dry weight (CDW), glycolipids, acetate, and ammonium concentration course. Error bands/bars indicate deviation from the mean (n = 2). Cultivation conditions: modified ONR7a medium, 3 L stirred-tank bioreactor, T = 30°C, pH = 7.3, N = 300–1,200 min^-1^ (cascaded), DO = 30%, F_Air_ = 32.4 L h^-1^, OD_start_ = 0.2, V_L_ = 1.2 L, 10 g L^-1^ acetate.

The batch fermentation (Figure 8) was started with 10 g L^-1^ (0.34 Cmol L^-1^) acetate at a filling volume of 1.2 L and performed for 49 h. Initially, the OTR remained constant for 6 h during the early stage of cultivation and then gradually declined until 16 h during the lag phase. Starting from 16 h, the OTR exhibited a continuous increase until the end of cultivation, reaching a maximum OTR of 17.0 mmol L^-1^ h^-1^ (Figure 8A). This increase was due to exponential growth, comparable to shake flask cultivations (Section 3.5). The RQ was around 1.0 during the exponential phase due to the acetate growth without the production of significant side products. Following the lag phase, exponential growth occurred between 16 and 49 h, corresponding to the OTR data. The maximum CDW achieved was 3.0 g L^-1^, with a µ_max_ of 0.12 h^-1^, similar to shake flask cultivation (Figure 8B). The volumetric substrate uptake rate (r_s_) was 0.20 g L^-1^ h^-1^, and the maximum specific uptake rate (q_s,max_) was 0.50 g g^-1^ h^-1^ (8.3 mM g^-1^ h^-1^). The biomass-to-substrate yield (Y_X/S_) was determined to be 0.30 g g^-1^. The ammonium concentration remained at 0.1 g L^-1^ at the end of the cultivation. The biomass-to-ammonium yield (Y_X/N_) for acetate as a carbon source was 6.2 g g^-1^. The concentration of glycolipids increased proportionally with the CDW, reaching a peak of 43 mg L^-1^, 1.9-fold higher than that observed in shake flask cultivation. The elevated glycolipid titer in the stirred-tank bioreactor may be attributed to the more stable cultivation conditions, as the pH was regulated at 7.3. This controlled environment likely provided more energy and carbon for glycolipid production. A Y_P/S_ of 129 mg Cmol^-1^ and a Y_P/X_ of 14 mg g^-1^ were obtained. Furthermore, volumetric productivity of 0.9 mg L^-1^ h^-1^ was achieved during the batch fermentation, comparable to the stirred-tank bioreactor cultivation with C_14_.

In summary, the cultivation with acetate in the stirred-tank bioreactor proceeded under controlled conditions with a higher glycolipid production. Furthermore, many different performance parameters could be determined, which significantly simplifies further optimization, especially with regard to fed-batch fermentations. Antifoaming agents were added moderately in this fermentation (0.1 mL Antifoam 204). However, this can become a challenge in fed-batch processes where much higher biomass (leading to higher OTR, thus also more intensive stirring and foam formation) and product concentrations can be achieved. Consequently, growth and product kinetics were monitored during cultivation using bubble-free aeration by a membrane module.

### 3.8 Bubble-free membrane aeration prevents foam formation and thus antifoaming agent addition

After acetate batch fermentation with bubble aeration and antifoaming agent addition, a static membrane module (BT Membrane Module static 2L, BioThrust GmbH, Aachen, Germany) for bubble-free aeration was tested. The filling volume was increased to 2 L, and the O_2_ concentration in the supply gas was automatically increased *via* the X_O2_ cascade to meet the oxygen demand of *A. borkumensis*. This changing oxygen concentration in the supply gas complicates the calculation of OTR, so the O_2_ and CO_2_ concentrations in the exhaust gas were plotted instead.

**FIGURE 9:**
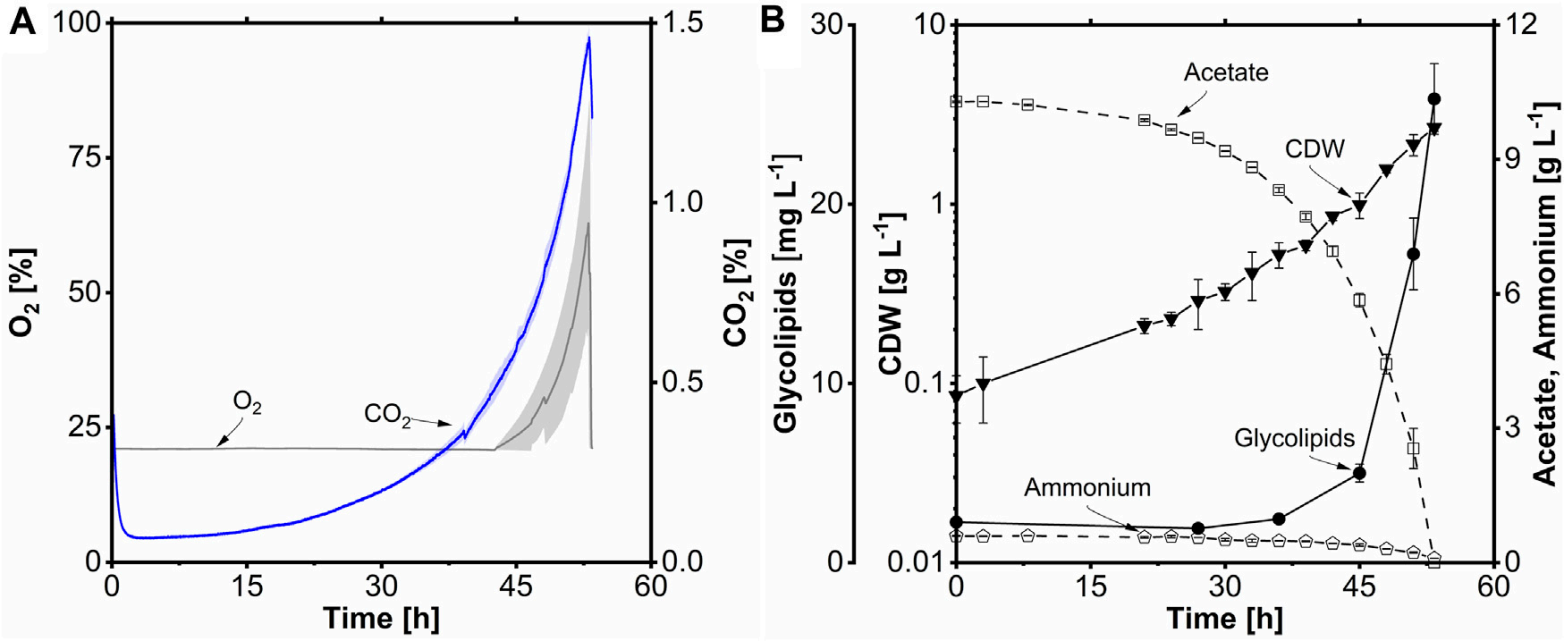
Membrane-aerated batch fermentation of *A. borkumensis* SK2 with acetate as carbon source. **(A)** Time course of O_2_ and CO_2_ volume concentration; **(B)** cell dry weight (CDW), glycolipid, acetate, and ammonium concentration course. Error bands/bars indicate deviation from the mean (n = 2). Cultivation conditions: modified ONR7a medium, 3 L stirred-tank bioreactor, BioThrust static membrane module 2 L, T = 30°C, pH = 7.3, N = 300 min^-1^, DO = 30%, F_Gas_ = 60 L h^-1^, TMP = 0.3 bar, X_O2_ = 21–100% (cascaded), OD_start_ = 0.2, V_L_ = 2.0 L, 10 g L^-1^ acetate.

After 15 h of lag phase, the carbon dioxide concentration increased exponentially, corresponding to a µ_max_ of 0.11 h^-1^ until the end of the cultivation at 53 h. After 46 h, the O_2_ concentration in the off-gas started to increase to 67% at the end of cultivation (Figure 9A). The increase can be explained by the DO cascade, which admixes pure O_2_ at a DO lower than 30%. Following the lag phase, exponential growth occurred between 16 and 53 h, mirroring the CO_2_ data from the off-gas. A maximum CDW concentration of 2.7 g L^-1^ was achieved (Figure 9A). Again, the lower biomass is most likely due to biofilm formation on the membrane. The r_s_ was 0.19 g L^-1^ h^-1^, and the q_s,max_ was 0.5 g g^-1^ h^-1^. The Y_X/S_ was determined to be 0.26 g g^-1^. The ammonium concentration remained at 0.1 g L^-1^ at the end of the cultivation, similar to the bubble-aerated process. The Y_X/N_ was 5.4 g g^-1^. The glycolipid concentration at the end of the cultivation was 26 mg L^-1^, 0.4-fold lower than in the bubble-aerated process. The lower glycolipid titer and the elongated process could be due to biofilm formation in the membrane process (Supplementary Figure S5). The membrane material (PMP) is very hydrophobic; the most hydrophobic cells are probably attached to the membrane module, which means the cells with the highest amount of glycolipid. Biofilm formation on hydrophobic surfaces, such as *n*-alkanes, is a well-known phenomenon for *A. borkumensis* and has often been reported in the literature (Naether et al., 2013; Abbasi et al., 2018; Godfrin et al., 2018). A Y_P/S_ of 78 mg Cmol^-1^ and a Y_P/X_ of 10 mg g^-1^ were obtained. Furthermore, volumetric productivity of 0.5 mg L^-1^ h^-1^ was achieved during batch fermentation.

Taken together, the results of the membrane-aerated batch fermentation using the static membrane module show that bubble-free aeration for glycolipid production without oxygen limitation, foam formation, and the addition of an antifoaming agent is possible. Bubble-free aeration could be an advantage in fed-batch processes, where high antifoaming agent additions are often required in biosurfactant-producing fermentations (Beuker *et al.* (2016) >15 g L^-1^ of antifoaming agent) since simultaneous high cell density leads to strong emulsion formation and thus to more difficult purification of the product (Demling et al., 2020). One disadvantage is biofilm formation on the membrane by *A. borkumensis*. However, this could possibly be further reduced with a dynamic membrane module, for example, the membrane stirrer by Bongartz *et al.* (2023), as this significantly increases the flow velocity and the shear rate of the membrane module and thus makes biofilm formation less likely.

## 4 Discussion

The marine bacterium *A. borkumensis* SK2 possesses several remarkable features. These include its ability to grow exclusively on various hydrocarbons as its sole carbon source, the production of a biosurfactant, and its capacity to synthesize and secrete storage lipids (Passeri et al., 1992; Abraham et al., 1998; Yakimov et al., 1998; Schneiker et al., 2006; Manilla-Pérez et al., 2010b). To optimize the cultivation of *A. borkumensis* SK2 for enhanced growth performance and glycolipid production, we considered various factors such as medium composition, temperature, and the spectrum of hydrophilic and hydrophobic substrates using different online analytics and state-of-the-art HPLC analytics for high-quality physiology. Furthermore, the challenges posed by the used substrates were investigated and addressed.

In many published studies, *A. borkumensis* SK2 cultivations are presented with challenges regarding recording physiological data, exhibiting suboptimal growth. We were able to confirm these insights in our study. The existing methods for measuring biomass are unsuitable, especially for hydrocarbon cultivation, because the hydrocarbons affect the optical density measurement due to emulsion formation during cultivation or adhere to the cells during CDW measurement, thus affecting the weight (Demling et al., 2020). Furthermore, biofilm formation around oil droplets makes sampling difficult (Prasad et al., 2022). This study solved this measuring issue by using OTR measurements. This parameter describes the metabolic activity of a culture in shake flasks or stirred-tank bioreactors, which is much more reliable. The fact that these challenges regarding physiological data have not been tackled before is probably because many studies are more concerned with fundamental research, especially bioremediation, alkane metabolism, and storage molecule formation (Golyshin et al., 2003; Hara et al., 2003; Sabirova et al., 2006a; Kalscheuer et al., 2007; Manilla-Pérez et al., 2010b; Manilla-Pérez et al., 2011; Naether et al., 2013; Bookstaver et al., 2015; Hassanshahian and Cappello, 2015; Gregson et al., 2019), while we focused on the application of the remarkable bacterium *A. borkumensis* for biosurfactant production. High-quality physiological insights are thus essential.

Most marine environments have low concentrations of inorganic nutrients, such as phosphate or nitrogen, and therefore often have high carbon-to-nitrogen or carbon-to-phosphate ratios, or both, which are unfavorable for microbial growth (Leahy and Colwell, 1990; Dashti et al., 2015). The ONR7a medium (Dyksterhouse et al., 1995) is an artificial seawater mineral salt medium. Therefore, it is designed to mimic seawater in that all major cations and anions are present at concentrations >1 mg L^-1^. The proportions of phosphate (89 mg L^-1^ Na_2_HPO_4_) and ammonium (0.27 g L^-1^ NH_4_Cl) were too low for high growth rates and biomass concentrations, essential for efficient bioreactor cultivations. These conditions with a high carbon-to-nitrogen ratio tend to promote the formation of polyhydroxyalkanoates, triacylglycerides, or wax esters in *A. borkumensis* SK2, so-called storage molecules rather than growth or biosurfactant production (Sabirova et al., 2006a; Kalscheuer et al., 2007; Manilla-Pérez et al., 2010a). Furthermore, these storage molecules are undesirable by-products in biosurfactant-producing cultures because they are produced from the same precursor molecules from the *de novo* fatty acid synthesis, lowering the potential yield on substrate.

Another factor addressed in this study that has not been sufficiently considered in previous studies during *A. borkumensis* cultivation is the pH value. During the cultivation with acids as carbon sources (pyruvate, acetate), the pH increases, and during cultivation with *n*-alkanes, the pH decreases due to the fatty acid intermediates formation during alkane degradation (Rojo, 2009; Cui et al., 2022). To address this issue in small-scale cultivations, the most straightforward approach is to use buffers with the disadvantage of increasing the osmotic pressure of the medium. (Kensy et al., 2009). The behavior of microorganisms and the formation of by-products, product yield, and biomass development can vary significantly when different microorganisms or media are employed. Susceptible microorganisms, like *Escherichia coli*, can cease growth if the pH drops below a critical threshold of approximately 5.0 (Losen et al., 2004).

Since pyruvate is the standard hydrophilic substrate for *A. borkumensis* in many studies, it was investigated in more detail in shake flasks in this study. It was found that pyruvate is unstable in ONR7a medium and dimerizes to parapyruvate (Chang et al., 2018). While we have found no evidence that this impedes growth (the resulting biomass at the end of the cultivation from 10 g L^-1^ pyruvate was similar to that when using 10 g L^-1^ acetate), the accurate determination of rates and yields was impossible, which is essential for designing an efficient bioprocess. Therefore, other hydrophilic carbon sources were investigated, but no new ones could be discovered, except for the already published carbon sources acetate and propionate (Yakimov et al., 1998). Since acetate is a sustainable carbon source that can be produced from lignocellulose, syngas, or CO_2_ and methyl formate, acetate was selected for closer examination (Kantzow et al., 2015; Ehsanipour et al., 2016; Jürling-Will et al., 2022). Thus, using bio-based acetic acid as a carbon source contributes to establishing a circular bioeconomy. Acetate is converted to acetyl-CoA *via* acetyl-CoA synthetase (AcsA) and is fed directly into the central carbon metabolism (Kiefer et al., 2021). The glyoxylate shunt holds particular significance in this context as it bypasses two decarboxylation steps and preserves two carbon skeletons for gluconeogenesis and biomass production, which is highly upregulated in *A. borkumensis* and thus the key metabolic intermediate in acetyl-CoA-grown cells is malate, formed through channeling of acetyl-CoA into the glyoxylate shunt (Sabirova et al., 2006b). Acetyl-CoA is the direct precursor molecule to the *de novo* fatty acid synthesis (Grahame Hardie, 1989), which is required to produce the glycolipid of *A. borkumensis* (Passeri et al., 1992; Cui et al., 2022). However, acetate can inhibit bacterial growth in its protonated form, which dissociates in the slightly alkaline cytosol. Consequently, this leads to cytosol acidification, disrupting the transmembrane pH gradient required for ATP synthesis through the proton motive force (Baronofsky et al., 1984; Axe and Bailey, 1995). The growth inhibition resulting from this phenomenon can lead to an extended lag phase, reduced biomass yield, and lower product yield during the fermentation process (Takahashi et al., 1999). A prolonged lag phase and reduced biomass yield with increasing acetate concentrations, but not higher than 15 g L^-1^, was also observed in this study (Figure 5B; Figures 6A,B). Several other bacteria, including *P. putida*, *E. coli*, and *Corynebacterium glutamicum*, have demonstrated biotechnological production of different products using acetate, with the ability to grow on acetate concentrations of at least 10 g L^-1^ (Noh et al., 2018; Arnold et al., 2019; Wolf et al., 2020; Schmollack et al., 2022). Acetate serves as a crucial precursor for various biotechnological products, such as itaconic acid production in *E. coli* and *C. glutamicum*, as well as rhamnolipid production in *P. putida* (Noh et al., 2018; Arnold et al., 2019; Merkel et al., 2022).

In the TOM shaker experiment (Figure 6), consistently higher growth rates of *A. borkumensis*, when grown on pyruvate (µ_max_ = 0.25 h^-1^) or acetate (µ_max_ = 0.16 h^-1^), were observed, compared to *n*-hexadecane (µ_max_ = 0.09 h^-1^). Previously, Naether *et al.* (2013) reported a higher growth rate on *n*-hexadecane than on pyruvate, possibly due to the above-described effects in *n*-hexadecane cultivations. In contrast, our results align with those of Barbato *et al.* (2016), who found a higher cell density (measured by flow cytometry) on pyruvate than on *n*-dodecane. Furthermore, Naether *et al.* (2013) reported that the µ_max_ increases with increasing chain length (up to C_18_) of the *n*-alkanes. In our study, however, the lag phase increased for the *n*-alkanes, and the µ_max_ decreased with increasing chain length. In the aqueous environment, the uptake and degradation rates of various organic compounds by microbial populations are typically proportional to the concentration of the compound, following Michaelis-Menten kinetics (Boethling and Alexander, 1979). This behavior has been demonstrated for toluene, a low-molecular-weight aromatic hydrocarbon with relatively high water solubility (520 mg L^-1^), but may not apply to more insoluble hydrocarbons (Button and Robertson, 1986). For higher-molecular-weight aromatic hydrocarbons like naphthalene and phenanthrene, their rates of degradation are associated with water solubilities rather than total substrate concentrations (Thomas et al., 1986; Abbasian et al., 2015). Water solubility decreases with the increasing chain length of the *n*-alkanes. In the case of long *n*-alkanes with water solubilities less than 0.01 mg L^-1^ (Bell, 1973), microbial degradation occurs at rates surpassing the solubility rates of hydrocarbons (Thomas et al., 1986; Abbasian et al., 2015). These degradation rates depend on the available hydrocarbon surface area for emulsification or cell physical attachment (Nakahara et al., 1977).

In this study, the glycolipid titer was highest with tetradecane at 66 mg L^-1^ in shake flask experiments, followed by hexadecane and acetate at 51 and 23 mg L^-1^, respectively. There are not many data sets in the literature on glycolipid production in *A. borkumensis*. The initial production of this glycolipid dates back to 1992. After nitrogen-limited fed-batch fermentation, a titer of 1.7 g L^-1^ of glycolipid and a specific yield of 70 mg g_CDW_^-1^ was achieved (Passeri et al., 1992). The supposedly high glycolipid titer was most likely reached since a fed-batch was performed, *i.e.*, significantly more carbon was introduced into the system, and a different nitrogen source (NaNO_3_) was used. Studies in shake flask experiments of *P. aeruginosa* have also shown that compared to NH_4_Cl, switching to NaNO_3_, urea, or NH_4_NO_3_ resulted in improved rhamnolipid production (Saikia et al., 2012; Moussa et al., 2014). However, glycolipid concentration has been determined *via* thin-layer chromatography with simultaneous spot intensity measurement (Passeri et al., 1992). Without an authentic standard and high sample purity, this method is semi-quantitative, and an HPLC-based method is significantly more accurate. The quantification of biosurfactants is often a problem within the biosurfactant literature because the samples are usually complex, and there is often a lack of pure standard substances. Here, we used an HPLC system with an inverse gradient and a charged aerosol detector that allows quantification even without a standard substance of the glycolipid (Lipphardt et al., 2023).

In Passeri et al. (1992) only the production of the glycolipid consisting of four 3-hydroxy-fatty acids with different chain lengths and one glucose molecule was shown. However, a recent study by Cui *et al.* (2022) demonstrated that the glycine-free form does not exist and that the glycine-glucolipid is localized at the cell membrane. Other studies that measured increased cell surface hydrophobicity supported this membrane association of the glycine-glucolipid (Naether et al., 2013; Abbasi et al., 2018; Godfrin et al., 2018). The specific yields (Y_P/X_) are consistent with our study as more as twice as much glycolipid is formed per biomass with hexadecane (17 mg g_CDW_^-1^) than with pyruvate (6 mg g_CDW_^-1^) (Cui et al., 2022). However, the glycolipid titer with hexadecane as a carbon source was reported to be 0.4-fold lower than with pyruvate. The cultures in the study of Cui *et al.* (2022) were probably oxygen-limited since they were agitated only at 180 rpm with a filling volume of 20%. It is usually recommended to perform shake flask cultivations with a filling volume of 5%–10% and higher shaking speeds to exclude oxygen limitation (Anderlei and Büchs, 2001). As Schlosser *et al.* (2021) mentioned, the oxygen transfer rate can impact biosurfactant production. Additionally, the reduced mixing of the second phase further contributes to slower and incomplete degradation, consequently affecting biosurfactant production.

As with glycolipid, there are only a few studies regarding fermentations in the stirred-tank bioreactor with *A. borkumensis*. To the best of our knowledge, only two other research groups cultivated *A. borkumensis* in a stirred-tank bioreactor and produced a product simultaneously (Passeri et al., 1992; Kadri et al., 2018). Kadri *et al.* (2018) used hexadecane or motor oil as a carbon source and produced an alkane hydroxylase and a lipase for enzymatic biodegradation of contaminated groundwater. Again, the cultivation conditions do not appear to be optimal, as only an OTR_max_ of circa 1 mmol L^-1^ h^-1^ was achieved with 30 or 50 g L^-1^ oil as a carbon source. This study reported an OTR_max_ of 8.6 mmol L^-1^ h^-1^ with 4.8 g L^-1^ tetradecane, which is 8.6-fold higher than in the previously mentioned study, although significantly less carbon was used. Furthermore, a glycolipid titer of 53 mg L^-1^ was achieved, with a Y_P/S_ of 155 mg Cmol^-1^. The titer is 0.2-fold lower than in the C_14_ shake flask (66 mg L^-1^). Nevertheless, the STY is 1.7-fold higher due to a higher mass transfer rate due to the more efficient mixing in the stirred-tank bioreactor and the consequent higher surface-to-volume ratio of the oil droplets.

One of the challenges encountered when producing biosurfactants using oils as a carbon source and antifoaming agents in stirred-tank bioreactors is the formation of emulsions. Downstream processing approaches often struggle to handle these emulsions effectively (Brandenbusch et al., 2010; Janssen et al., 2023). Additionally, hydrophobic substrates typically incur higher costs, posing a notable disadvantage, particularly when competing with well-established, cost-effective production processes (Chong and Li, 2017). To circumvent these challenges, batch fermentations with bubble aeration and bubble-free membrane aeration were performed using acetate as the carbon source. As shown in other publications with other microorganisms and cell lines, the batch fermentation with bubble-free membrane aeration showed no foam formation, so antifoaming agent addition was unnecessary (Frahm et al., 2009; Coutte et al., 2010; Bongartz et al., 2021; Bongartz et al., 2023).

In summary, this study reports glycolipid production with *A. borkumensis* SK2 using different carbon sources and high-quality physiology data using state-of-the-art online growth and HPLC glycolipid analytics, which enabled efficient stirred-tank bioreactor cultivations.

## Data Availability

The original contributions presented in the study are included in the article/Supplementary Material, further inquiries can be directed to the corresponding author.
