# Supplementary material for: High-quality physiology of Alcanivorax borkumensis SK2 producing glycolipids enables efficient stirred-tank bioreactor cultivation
Source: Front Bioeng Biotechnol. 2023 Nov 23;11:1325019. doi: 10.3389/fbioe.2023.1325019 (PMC10710537; doi:10.3389/fbioe.2023.1325019)
Supplement: Supplementary file 1 [file DataSheet1.docx]

Supplementary Material

High-quality physiology of *Alcanivorax borkumensis* SK2 producing glycolipids enables efficient stirred-tank bioreactor cultivation

Tobias Karmainski^1^, Marie R. E. Dielentheis-Frenken^1^, Marie K. Lipa^1^, An N. T. Phan^1^, Lars M. Blank^1^, Till Tiso^1*^

^1^iAMB – Institute of Applied Microbiology, ABBt – Aachen Biology and Biotechnology, RWTH Aachen University, Aachen, Germany

*** Correspondence:**Till Tiso
till.tiso@rwth-aachen.de

# Supplementary Figures

#



# Supplementary Figure 1. Growth Profiler cultivation of modified ONR7a with different phosphate concentrations with *A. borkumensis* SK2 on 10 g L^-1^ pyruvate (n = 3). Cultivation conditions: modified ONR7a medium, 24-well white plate, N = 225 rpm, T = 30°C, OD_start_ = 0.2, V_L_ = 1.0 mL.

#



# Supplementary Figure 2. Growth Profiler cultivation of modified ONR7a with different nitrogen concentrations with *A. borkumensis* SK2 on 10 g L^-1^ pyruvate (n = 3). Cultivation conditions: modified ONR7a medium, 24-well white plate, N = 225 rpm, T = 30°C, OD_start_ = 0.2, V_L_ = 1.0 mL.


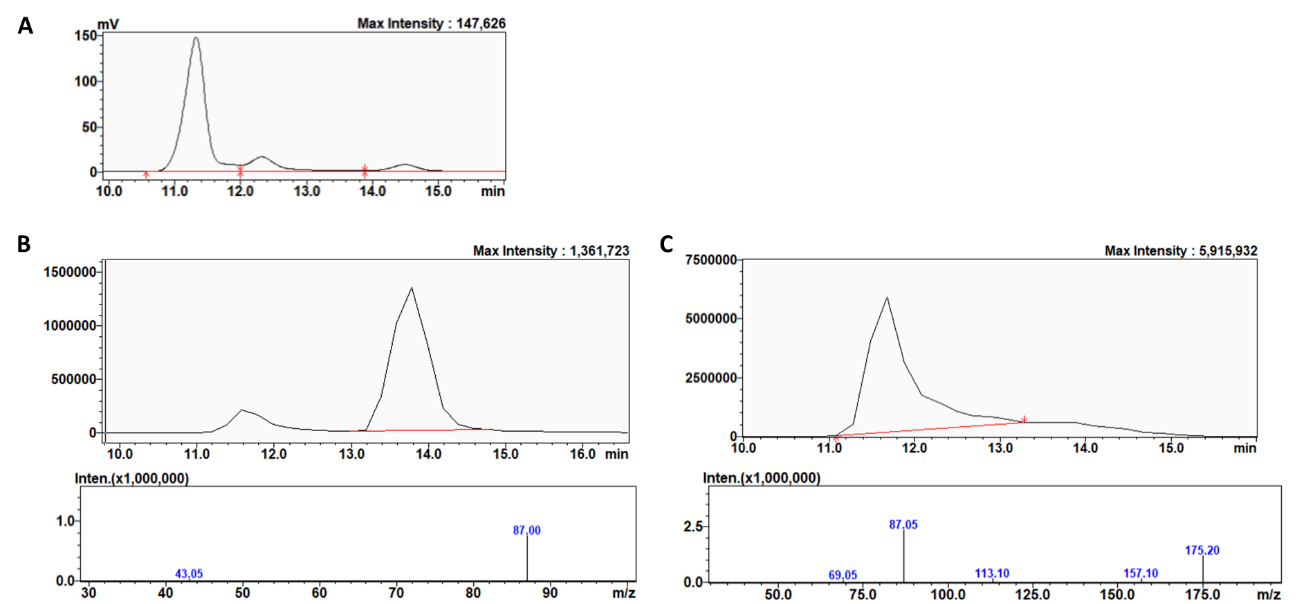


**Supplementary Figure 3.** The pyruvate cultivation sample of *A. borkumensis* SK2 was analyzed with LC-UV/RI-MS^2^. **(A)** HPLC chromatogram from RI detector. Chromatogram (upper panel) and mass spectrum (lower panel) obtained in product ion scan of m/z 87 **(B)** and m/z 175.2 **(C)**.


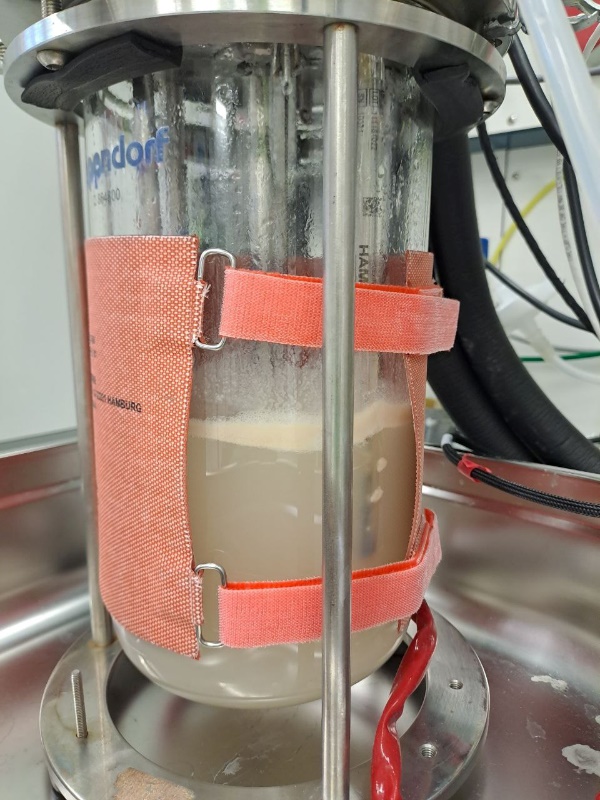


**Supplementary Figure 4.** Exemplary picture of the bioreactor during the *n*-tetradecane batch fermentation. Biofilm formation at the vessel wall and cell flocculation at the pH probe can be seen.


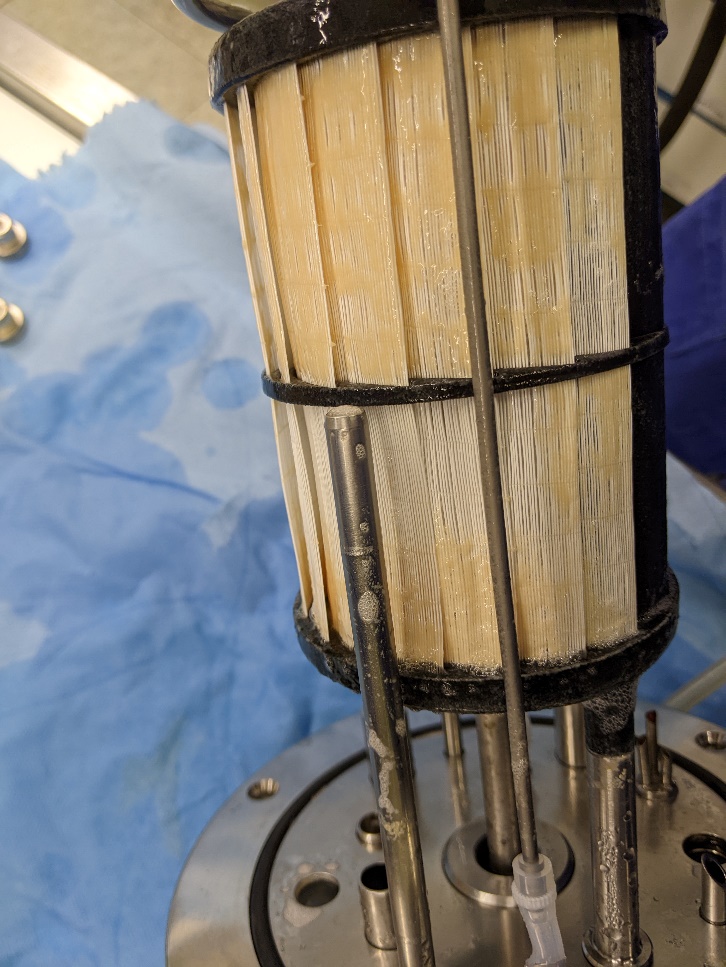


**Supplementary Figure 5.** Exemplary picture of the bioreactor installation at the end of the acetate batch fermentation with the static membrane module for bubble-free aeration. Biofilm formation on the membrane surface can be seen.
